# Association between Urinary Phthalate Metabolite Concentration and Atopic Dermatitis in Korean Adolescents Participating in the Third Korean National Environmental Health Survey, 2015–2017

**DOI:** 10.3390/ijerph18052261

**Published:** 2021-02-25

**Authors:** Sang-Woo Kim, Jeongho Lee, Soon-Chan Kwon, June-Hee Lee

**Affiliations:** 1 Department of Occupational and Environmental Medicine, Soonchunhyang University Hospital, 59 Daesagwan-ro, Yongsan-gu, Seoul 04401, Korea; 125155@schmc.ac.kr; 2 Department of Pediatrics, Soonchunhyang University Hospital, 59 Daesagwan-ro, Yongsan-gu, Seoul 04401, Korea; ljh@schmc.ac.kr; 3 Department of Occupational and Environmental Medicine, Soonchunhyang University Cheonan Hospital, 31 Suncheonhyang 6-gil, Dongnam-gu, Cheonan-si 31151, Korea; sckwon@sch.ac.kr

**Keywords:** adolescents, atopic dermatitis, urinary phthalate metabolites

## Abstract

Previous studies have highlighted the potential health effects of phthalate exposure, such as atopic dermatitis and asthma. However, evidence supporting the association between phthalate exposure and atopic dermatitis is limited and based on data collected from Western populations. This study aimed to analyze the association between phthalate exposure and atopic dermatitis in Korean adolescents aged 12–17 years using a nationally representative dataset. We conducted a cross-sectional study using a publicly available dataset from the third Korean National Environmental Health Survey (*n* = 797). We divided the study participants into four quartiles according to urinary phthalate metabolite concentrations. The odds ratio of having atopic dermatitis was calculated using the first quartile as the reference group in binary logistic regression. We found that in the logistic regression model, both the urinary Mono-(2-ethyl-5-carboxypentyl) phthalate (MECCP; OR: 1.81; CI: 1.01–3.25) and Mono-benzyl phthalate (MBzP; OR: 1.81; CI: 1.01–3.25) concentrations in the highest quartile were positively associated with atopic dermatitis. The atopic dermatitis group had a significantly higher mean urinary MECCP and MBzP concentration. In the future, longitudinal studies involving repeated measurements are warranted to analyze the long-term effects of phthalate.

## 1. Introduction

Phthalates are used as plasticizers in industrial plastic products including medical devices, packaging materials, dolls, and other toys [1]. Currently, phthalate ester is the most commonly used plasticizer in the production of polyvinyl chloride (PVC), which is a commonly used thermoplastic material [2]. As phthalates are now ubiquitous in the environment, people are regularly exposed to these chemicals through various routes, particularly dietary intake through contaminated food or water sources, dermal absorption through cosmetics or personal hygiene products, and aerosol inhalation through hair spray or nail polish [3].

Exposure to phthalates is can have adverse effects on the male reproductive system. For example, phthalates such as di-2-ethylhexyl phthalate (DEHP), dibutyl phthalate (DBP), and benzylbutyl phthalate (BzBP) are known to exert anti-androgenic effects, which disrupt the male reproductive system by reducing testosterone production in fetal testes [4,5,6]. 

In addition to these effects on the male reproductive system, several studies have reported the possibility of additional deleterious health effects, such as allergic diseases because of phthalate exposure [7,8,9]. Several hypotheses have been proposed on the occurrence or exacerbation of allergic disease by phthalates. For example, according to previous studies, pulmonary phthalate exposure is known to be involved in the activation of peroxisome proliferation activated receptors (PPAR) in the lung, which may be associated with developing or worsening of asthma [10]. In addition, an experimental study using human epithelial cell lines demonstrated increased levels of pro-inflammatory cytokines (IL-6 and IL-8) due to the adjuvant effect of monophthalates such as mono-n-butyl phthalate (MnBP) and mono-benzyl phthalate (MBzP) [11]. Another study showed that DEHP enhanced the production of inflammatory cytokines and chemokines in macrophages, which led to exacerbation of the inflammatory responses [12].

Recent human epidemiologic studies have also supported an association between exposure to certain kinds of phthalates and allergic diseases. Chinonso et al. (2012) examined an association between urinary phthalate metabolites and current asthma in adults and children who took part in the National Health and Nutrition Examination Survey (NHANES) 2007–2012. They found that MBzP levels were positively associated with the prevalence of self-reported asthma in children [13]. In a time-series study targeting 3–7-year-old children with atopic dermatitis, increased urinary mono-isobutyl phthalate (MiBP) levels were associated with aggravation of atopic dermatitis symptoms [14]. In contrast, Vernet et al. (2017) showed that low- and high-molecular-weight phthalate did not exhibit any clear association with increased asthma rates and several respiratory outcomes [15].

Although an increasing number of studies have demonstrated an association between phthalate exposure and allergic disease, the result of each study is controversial and most previous studies involving the bio-monitoring data have been based on data from Western populations. In Korea, the National Institute of Environmental Research (NIER) has been collecting bio-monitoring data since 2009 with the intention to determine the level of public exposure to harmful environmental substances. The third Korean National Environmental Health Survey (KoNEHS) was designed to represent the whole Korean population, and children younger than 19 years of age began to be included since 2015. 

Atopic dermatitis is a chronic and recurrent inflammatory skin disease characterized by pruritus, dry skin, and characteristic eczema, and it is most often the first manifestation of the allergic march that results in asthma and allergic rhinitis [16]. Therefore, in this study we conducted the first analysis of the association between phthalate exposure and atopic dermatitis among Korean adolescents using a nationally representative dataset from the KoNEHS. We expect that our bio-monitoring data will be comparable with current Western data and hope that our study will provide a foundation for larger studies on this topic in the Korean population in the future.

## 2. Materials and Methods 

### 2.1. Study Population

In this cross-sectional study, we used a publicly available dataset from the third KoNEHS, conducted during 2015–2017, to assess the relationship between phthalate exposure and atopic dermatitis among Korean adolescents. We excluded children under 12 years of age, as blood samples were not collected from them. Accordingly, this study included middle and high school students aged 12–17 years who completed a questionnaire pertaining to allergic disease and provided blood and urine samples. To ensure that the included urine samples had been analyzed accurately, subjects deemed outliers with respect to the normal creatinine range (0.3–3.0 mg/dL) were excluded from our study analysis. Finally, 797 subjects were included in our study. 

### 2.2. Atopic Dermatitis, Total Immunoglobulin E (IgE), and Phthalate Metabolites

In the KoNEHS dataset, self-reported questionnaires and serum total IgE concentrations were used to evaluate the morbidity and disease activity of atopic dermatitis. To assess three types of allergic diseases (atopic dermatitis, asthma, and allergic rhinitis), we referred to the self-reported questionnaires on when the respondent was first diagnosed, the presence of current symptoms, and the current treatment status. 

The concentration of serum total IgE was measured with a photometer, using direct chemiluminescence, during a competitive immunoassay. The antigen-antibody reaction was carried out by incubating the sample and alkaline phosphatase-conjugated antibodies on beads coated with total IgE-specific antibodies for 30 min at 37 °C. The chemiluminescent substrate was allowed to undergo a luminescent reaction for 5 min in the presence of alkaline phosphatase, and the degree of luminescence was measured with a photometer and used to calculate the sample’s concentration. 

The concentration of urinary phthalate metabolites was analyzed by liquid chromatography mass spectrometry (LC-MS), and electrospray ionization (ESI) was used as the ionization method. The sample was hydrolyzed with β-glucuronidase/aryl sulfatase and then extracted with ethyl acetate. Thereafter, the concentration of the urinary phthalate metabolite was calculated using a calibration curve prepared by standard addition, wherein a certain amount of standard solution was added to the sample. Eight phthalate metabolites, including three DEHP metabolites (mono-(2-ethyl-5-hydroxyhexyl) phthalate (MEHHP), mono-(2-ethyl-5-oxohexyl) phthalate (MEOHP), and mono-(2-ethyl-5-carbopentyl) phthalate (MECPP)), were analyzed in this study (Table 1). The values were adjusted to the urine creatinine concentration. The urine phthalate metabolites and serum total IgE concentration data were positively skewed; hence, the geometric means of these data were compared between the atopic dermatitis and non-atopic dermatitis groups after natural log transformation.

### 2.3. Statistical Analysis

All statistical analyses were conducted in consideration of the complex sample design of the third KoNEHS. We used urinary creatinine-corrected phthalate metabolite concentrations in the statistical analyses. Covariate data were collected using self-reported questionnaires or anthropometric measurements. BMI (Body mass index) (kg/m^2^) was calculated by dividing the measured weight (kg) by the square of the measured height (m). Our study sample comprised of middle and high school students; hence, we classified the BMI values according to the 2017 growth chart for Korean children and adolescents developed by the Korea Centers for Disease Control and Prevention (KCDC). Subjects with a BMI below the fifth percentile, within the fifth to the 85th percentile, above the 85th percentile, and above the 95th percentile were classified as underweight, normal weight, overweight, and obese, respectively. Using the questionnaire responses, subjects’ economic statuses were classified as monthly incomes less than two million won, two to five million won, and more than five million won. Secondhand smoke exposure was also categorized as no exposure, fewer than five exposures per week, and more than five exposures per week, according to the self-reported questionnaires. The presence of current allergic diseases, current symptoms, and the treatment statuses of patients with atopic dermatitis, asthma, and allergic rhinitis were also evaluated using self-reported questionnaires. 

The subjects were divided into atopic dermatitis and non-atopic dermatitis groups, as shown in Table 2. The association of atopic dermatitis with each variable was analyzed using a chi-square test. *p*-value ≤ 0.05 was considered statistically significant. We have represented information on the eight phthalate metabolites included in this study in Table 1. We compared the geometric mean of the urinary creatinine-corrected phthalate metabolite concentrations between the atopic dermatitis and non-atopic dermatitis group using a *t*-test in Table 3. We used binary logistic regression models to estimate the associations between urinary phthalates and atopic dermatitis. We also expanded our models to include a group of subjects diagnosed with atopic dermatitis and who had abnormal total IgE concentration (≥100 IU/mL) to evaluate associations between urinary phthalates and the current disease activity of atopic dermatitis. In the logistic regression model, the urine concentrations of the phthalate metabolites were divided into four quartiles. The odds ratios (ORs) and 95% confidence intervals (CIs) were calculated using the first quartile as the reference group (Table 4 and Table 5). ORs and 95% CIs were calculated using both crude and multivariate logistic regression models after adjusting for potential covariates (Table 5).

## 3. Results

Information on the eight phthalate metabolites included in this study is shown in Table 1. We have provided the abbreviations and major uses of five phthalate diesters and their eight monoester metabolites in the table.

Table 2 presents data of the general characteristics and current allergic disease statuses of the subjects in the atopic dermatitis and non-atopic dermatitis groups. The two groups did not differ significantly with respect to gender, school grade, BMI, household income, and secondhand smoke exposure.

Among the subjects with atopic dermatitis (*n* = 249), 93 (37.3%) had skin lesions and 30 (12.0%) were currently receiving treatment for this condition. Subjects in the atopic dermatitis group had a significantly higher incidence of other allergic diseases than those in the non-atopic dermatitis group. For example, 15.7% of subjects in the atopic dermatitis group had asthma, and this rate was more than threefold higher than that in the non-atopic dermatitis group (4.4%). The rates of allergic rhinitis were 57% and 30.1% in the atopic dermatitis and non-atopic dermatitis groups, respectively. Moreover, 45.4% and 22.3% of subjects in the atopic dermatitis and non-atopic dermatitis groups, respectively, reported of having current symptoms of allergic rhinitis, and this difference was significant (*p* < 0.001).

Table 3 presents the serum total IgE and urinary phthalate metabolite concentrations in the non-atopic dermatitis and atopic dermatitis groups. Notably, the geometric mean total IgE concentration in the atopic dermatitis group was 135.7 IU/mL, which was 1.5-fold higher than the concentration (88.2 IU/mL) in the non-atopic dermatitis group. Regarding the phthalate metabolites, the urine concentrations of both MECCP and MBzP differed significantly between the atopic dermatitis and non-atopic dermatitis groups. The MECCP concentrations in these groups were 19.13 μg and 17.27 μg, respectively. The MBzP concentrations were 2.05 μg/mL and 1.57 μg/mL, respectively. Both MECCP and MBzP concentrations were significantly higher in the atopic dermatitis group.

Logistic regression analyses revealed associations of urinary phthalate monoester metabolites with atopic dermatitis as shown in Table 4 and Table 5. In the crude model, urinary MECCP concentration in the highest quartile was positively associated with atopic dermatitis (OR: 1.77; CI: 1.14–2.75). This association did not change significantly in the multivariate model adjusted for the covariates of gender, school grade, household income, secondhand smoke exposure, and BMI (OR: 1.81; CI: 1.16–2.80). The fourth quartile of urinary concentration of MBzP was also positively associated with atopic dermatitis in both the crude model (OR: 1.76; CI: 1.02–3.03) and multivariate model (OR: 1.81; CI: 1.01–3.25). In the logistic regression model, which analyzed the association of atopic dermatitis with an abnormal serum total IgE concentration (>100 IU/mL), no significant associations were observed with MECCP in either model or with MBzP in the crude model (OR: 1.77; CI: 1.02–3.03). However, the multivariate model revealed a positive association between the fourth quartile of urinary MBzP concentration and atopic dermatitis with an abnormal serum total IgE (OR: 1.91; CI: 1.04–3.48).

## 4. Discussion

As the reported half-life of phthalates in a human body is less than 24 h and phthalate diesters are rapidly excreted in the urine as monoester metabolites, urinary metabolite concentrations are appropriate biomarkers for measuring the exposure of humans to the parent phthalate compounds [17]. Prior to the analysis, we compared the urinary concentrations of phthalate metabolites in our dataset with previous literature to assess the exposure levels of the Korean population. In previous biomonitoring studies, the exposure levels varied according to the study period and countries, but generally declined over time since the 2000s [18]. The results of our study are lower than the results of studies conducted in Europe in the early 2000s, but similar or higher than the results of recent years. In an analysis of participants in the 2005–2006 NHANES of the United States, the urinary concentrations of phthalate metabolites were higher than those reported in our study [19]. In European studies of young adults aged 19–29 years, the respective urinary MnBP and MBzP concentrations were 54.5 and 15.0 in 2011 but decreased to 1.3 and 1.3 in 2016, and these values were relatively lower than those observed in our study [20]. Data collected by the German Environmental Specialized Bank (ESB) during 1988–2015 revealed that the exposure levels in 2007 were similar to those in our study, but gradually decreased to a lower level over time [18].

Of the eight analyzed metabolites, only the DEHP metabolite MECCP and the BzBP metabolite MBzP were shown to be associated with atopic dermatitis in our study. DEHP accounts for approximately 50% of all phthalates used [21,22], and this chemical is widely used in the production of flexible PVC, clothing, toys, food containers, buildings, and household appliances [23]. In our analysis, the atopic dermatitis group had a significantly higher urinary MECCP and MBzP concentration than the non-atopic dermatitis group, and the fourth MECCP and MBzP quartile was positively associated with atopic dermatitis. 

Traditional epidemiological studies have demonstrated associations between high-molecular-weight (HMW) phthalates with allergic disease. In this study, all analyzed phthalates except MnBP were classified as HMW. In previous studies, DEHP and BzBP have been the most actively discussed phthalates with respect to allergic diseases, leading to the mechanistic hypothesis that these phthalates have an adjuvant effect on antigen-specific Ig production [24]. In a cross-sectional study of 2325 NHANES participants, Hoppin et al. revealed the associations of MBzP with allergy symptoms and specific IgE sensitization in adults [19]. Jacakola et al. (1999, 2000, 2004) reported an association of indoor PVC flooring with asthma and bronchial structure in children [25,26,27]. Both DEHP and BzBP and their monoester metabolites have been associated with asthma and wheezing in adults [19,28]. Studies of children have shown that exposures to DEHP, BzBP, DBP, and diethyl phthalate (DEP) during the gestational period were related to the incidence of allergic reactions in infants and toddlers. Takano et al. reported that in mice with atopic dermatitis, skin lesions were aggravated in response to the intraperitoneal injection of DEHP, as demonstrated by both macroscopic and microscopic in vitro evaluations [29]. Thor Larsen et al. (2001) also reported a meaningful increase in the production of antigen-specific IgG1 in rodents subjected to DEHP injection [30].

We did not observe a significant association between MECCP and atopic dermatitis when it was accompanied by total IgE abnormality, whereas MBzP was associated with an increased risk of atopic dermatitis in the high exposure quartile. Generally, the serum total IgE concentration is analyzed as a measure of allergic disease activity, as it tends to be higher in patients with atopic dermatitis. Moreover, the concentration of IgE is known to increase as the disease condition becomes exacerbated [24]. It is often very difficult to determine the appropriate level of total IgE concentration that would be useful for distinguishing allergic disease, as this variable is also affected by age and environmental and demographic factors [31]. Despite this limitation, a high serum total IgE concentration is a useful indicator of a positive specific IgE rate, a positive conversion of a skin prick test, and the risk of developing allergic diseases [32,33]. Therefore, because we limited our study subjects within a specific age group (12–17 years) and targeted our analysis to atopic dermatitis, we would expect that the serum total IgE concentration can provide information about the activity of atopic disease in this population.

Earlier studies based on phthalate bio-monitoring data mostly used single measurements to determine exposure levels. However, this approach was limited with respect to the assessment of long-term exposure, as most phthalates have a relatively short (<24 h) half-life in the body. We note that our study also has a cross-sectional design, and therefore it was difficult to directly obtain information about long-term exposure levels. However, humans are commonly exposed to phthalates during everyday life. Therefore, those who have a high exposure level from a single measurement are more likely to be vulnerable to phthalate exposure in their residential environment or through their diet. For example, a repeated analysis of first morning urine samples from Danish children revealed significant correlations between the daily measurements and with the indoor phthalate concentrations in the children’s residential bedrooms and daycare centers [34]. These results suggest that even single measurements can provide meaningful information about long-term environmental exposure. 

As we evaluated atopic dermatitis on the basis of self-reported questionnaires, the prevalence of atopic dermatitis in this study may have been underestimated or overestimated. According to previous studies, the prevalence of atopic dermatitis in industrialized countries was about 15–30%, and it tended to increase in recent decades [35]. The prevalence of atopic dermatitis in Korean adolescents in our study was about 31%. Although this result does not deviate significantly from the results of previous studies, it is still possible that it is overestimated compared to the prevalence reported by physicians. In addition, KoNEHS data have a limitation in that there is no detailed information on the severity of atopic dermatitis and other blood tests associated with allergic diseases. Therefore, follow-up studies considering the severity of atopic dermatitis and applying strict diagnostic criteria in the selection of subjects are needed for a more accurate analysis. 

In our study, we observed a significant association of atopic dermatitis only at the highest levels of exposure to certain phthalates, which suggests a dose–response relationship. This finding represents a possibility of a causal relationship between phthalate exposure and atopic dermatitis. Another notable strength of our study is that this is the first study to analyze the association between phthalate exposure and atopic dermatitis using nationally representative data from the Korean population. Hitherto, most existing bio-monitoring data regarding phthalate exposure have been derived from Western populations in Europe and the USA; hence, the findings of this study on the Korean population is useful for further studies on in topic in this population.

## 5. Conclusions

In conclusion, we have demonstrated a positive association between MBzP and MECCP and the development of atopic dermatitis in a sample of Korean adolescents. The cause of atopic dermatitis is not clear, but various factors including environmental, genetic, and immunological factors are known to be involved. In this study, we revealed the potential association of exposure to high concentrations of certain phthalates with atopic dermatitis using nationally representative data. Human biomonitoring studies are useful for determining the levels of exposure to environmentally harmful substances. In the future, longitudinal studies involving repeated measurements will be needed to analyze the long-term effects of phthalate to atopic dermatitis and overcome issues related to the short half-lives of phthalate metabolites.

## Figures and Tables

**Table 1:** Major phthalate diesters and their corresponding metabolites with major uses included in this study.

Phthalate Diesters	Abb.	Monoester Metabolite	Abb.	Major Uses
Di-2-ethylhexyl phthalate	DEHP	Mono-(2-ethyl-5-hydroxyhexyl) phthalate	MEHHP	PVC, plastics, medical equipment, and tubing
		Mono-(2-ethyl-5-oxohexyl) phthalate	MEOHP	
		Mono-(2-ethyl-5-carboxypentyl) phthalate	MECCP	
Dibutyl phthalate	DBP	Mono-n-butyl phthalate	MnBP	Polyvinyl emulsions, adhesives, and coatings
Benzylbutyl phthalate	BzBP	Mono-benzyl phthalate	MBzP	PVC, plastics, coatings, adhesives, and printing inks
Diisononyl phthalate	DiNP	Mono-carboxyoctyl phthalate	MCOP	PVC, inks, paints, and sealants
Diisodecyl phthalate	DiDP	Mono-carboxyisononyl phthatlate	MCNP	PVC, artificial leather, inks, packaging materials
Di-n-octyl phthalate	DnOP	Mono-(3-carboxypropyl) phthalate	MCPP	Polymer manufacturing, PVC, gloves, and flooring

Abb.: Abbreviation; PVC: Polyvinyl chloride.

**Table 2:** Demographic characteristics and current allergic status of the subjects in the atopic dermatitis and non-atopic dermatitis groups participating in Korean National Environmental Health Survey (KoNEHS) (2015–2017).

Variable	Total (*n* = 797)	Non-Atopic Dermatitis (*n* = 548)	Atopic Dermatitis (*n* = 249)	*p*-Value
	N (%)	N (%)	N (%)	
Gender				
Men	370 (46.4)	253 (46.2)	117 (47.0)	0.83
Women	427 (53.6)	295 (53.8)	132 (53.0)	
School Grade				
Middle school	407 (51.1)	274 (50.0)	133 (53.4)	0.372
High school	370 (48.9)	274 (50.0)	116 (46.6)	
BMI				
Underweight	22 (2.8)	12 (2.2)	10 (4.0)	0.288
Normal	574 (72.0)	402 (73.4)	172 (69.1)	
Overweight	87 (10.9)	55 (10.0)	32 (12.9)	
Obese	114 (14.3)	79 (14.4)	35 (14.1)	
Household Income (million won/month)				
<200	114 (14.3)	74 (13.5)	40 (16.1)	0.577
200–500	382 (47.9)	267 (48.7)	115 (46.2)	
>500	243 (30.5)	164 (29.9)	79 (31.7)	
Secondhand Smoke				
No	552 (69.3)	381 (69.5)	171 (68.7)	0.823
<5 times/week	186 (23.3)	125 (22.8)	61 (24.5)	
≥5 times/week	59 (7.4)	42 (7.7)	17 (6.8)	
Current Allergy Status				
Atopic dermatitis				
Diagnosis	249 (31.2)		249 (100.0)	
Current symptom	93 (11.7)		93 (37.3)	
Treatment	30 (3.8)		30 (12.0)	
Asthma				
Diagnosis	63 (7.9)	24 (4.4)	39 (15.7)	<0.001
Current symptom	9 (1.1)	4 (0.7)	5 (2.0)	<0.001
Treatment	4 (0.5)	1 (0.2)	3 (1.2)	<0.001
Allergic rhinitis				
Diagnosis	307 (38.5)	165 (30.1)	142 (57.0)	<0.001
Current symptom	235 (29.5)	122 (22.3)	113 (45.4)	<0.001
Treatment	52 (6.5)	18 (3.3)	34 (13.7)	<0.001

BMI: Body mass index.

**Table 3:** Total Immunoglobulin E (IgE) and urinary creatinine-adjusted concentration of phthalate metabolite for the atopic dermatitis and non-atopic dermatitis groups.

Analyte		GM	Min	25%	50%	75%	95%	Max
Total IgE Concentration (IU/mL)								
IgE	Non-atopic dermatitis	88.2 *	1.0	33.6	104.0	247.0	891.7	2000
	Atopic dermatitis	135.7 *	1.2	44.0	147.0	452.0	1877.6	2000
Urinary Phthalate Metabolites (μg/mL)								
MEHHP	Non-atopic dermatitis	8.63	0.30	5.95	8.64	13.12	25.85	119.39
	Atopic dermatitis	9.14	0.01	6.02	9.73	14.57	26.99	79.98
MEOHP	Non-atopic dermatitis	5.67	0.02	3.83	6.21	9.28	16.89	67.69
	Atopic dermatitis	6.16	0.61	3.79	6.54	10.82	20.09	59.75
MECCP	Non-atopic dermatitis	17.27 *	5.52	12.02	16.25	23.24	42.79	181.60
	Atopic dermatitis	19.13 *	4.16	13.41	18.32	26.76	51.36	107.26
MnBP	Non-atopic dermatitis	21.01	0.02	13.08	20.98	34.29	72.93	222.17
	Atopic dermatitis	23.55	2.70	13.30	21.03	41.69	97.28	254.96
MBzP	Non-atopic dermatitis	1.57 *	0.02	0.80	1.57	3.18	11.91	45.88
	Atopic dermatitis	2.05 *	0.02	0.98	1.90	4.24	14.52	99.62
MCOP	Non-atopic dermatitis	0.98	0.01	0.68	1.02	1.53	2.84	8.45
	Atopic dermatitis	1.15	0.01	0.77	1.17	1.64	3.27	31.25
MCNP	Non-atopic dermatitis	0.28	0.03	0.20	0.27	0.41	0.80	2.34
	Atopic dermatitis	0.3	0.03	0.21	0.29	0.43	0.87	1.98
MCPP	Non-atopic dermatitis	0.89	0.02	0.64	0.87	1.21	1.89	8.39
	Atopic dermatitis	0.93	0.02	0.68	0.94	1.32	2.15	4.36

* *p*-value < 0.05; IgE: Immunoglobulin E; GM: Geographic Mean; MEHHP: Mono-(2-ethyl-5-hydroxyhexyl) phthalate; MEOHP: Mono-(2-ethyl-5-oxohexyl) phthalate; MECCP: Mono-(2-ethyl-5-carboxypentyl) phthalate; MnBP: Mono-n-butyl phthalate; MBzP: Mono-benzyl phthalate; MCOP: Mono-carboxyoctyl phthalate; MCNP: Mono-carboxyisononyl phthatlate; MCPP: Mono-(3-carboxypropyl) phthalate; Min: Minimum; Max: Maximum.

**Table 4:** Association (OR (95% CI)) between urinary concentration of phthalate metabolites and atopic dermatitis according to the presence of elevation of total IgE (Crude model).

Quartile	Urinary Phthalate Metabolites							
	MEHHP	MEOHP	MECCP	MnBP	MBzP	MCOP	MCNP	MCPP
Atopic Dermatitis								
1 (ref)	1	1	1	1	1	1	1	1
2	0.74	1.06	1.04	0.96	1.38	1.05	0.99	1.07
	(0.41–1.33)	(0.70–1.61)	(0.71–1.54)	(0.54–1.73)	(0.79–2.41)	(0.64–1.71)	(0.59–1.65)	(0.63–1.83)
3	0.98	0.82	0.93	0.79	1.10	1.55	1.09	1.10
	(0.58–1.64)	(0.49–1.36)	(0.57–1.52)	(0.47–1.33)	(0.69–1.77)	(0.90–2.67)	(0.67–1.78)	(0.69–1.74)
4	1.41	1.48	1.77	1.25	1.76	1.33	1.11	1.24
	(0.85–2.33)	(0.94–2.34)	(1.14–2.75)	(0.70–2.22)	(1.02–3.03)	(0.79–2.23)	(0.69–1.78)	(0.75–2.07)
Atopic Dermatitis with Elevation of Total IgE								
1 (ref)	1	1	1	1	1	1	1	1
2	0.69	1.16	0.76	0.80	1.23	1.14	1.19	0.91
	(0.36–1.32)	(0.67–2.01)	(0.45–1.29)	(0.44–1.46)	(0.65–2.31)	(0.69–1.89)	(0.63–2.25)	(0.52–1.59)
3	0.61	0.67	0.63	0.83	0.87	1.30	1.31	1.34
	(0.34–1.08)	(0.36–1.23)	(0.32–1.24)	(0.46–1.52)	(0.48–1.55)	(0.73–2.31)	(0.75–2.29)	(0.82–2.20)
4	1.24	1.35	1.20	1.20	1.77	1.08	1.11	1.23
	(0.79–1.96)	(0.83–2.19)	(0.69–2.09)	(0.69–2.01)	(0.99–3.15)	(0.62–1.88)	(0.64–1.92)	(0.76–1.97)

MEHHP: Mono-(2-ethyl-5-hydroxyhexyl) phthalate; MEOHP: Mono-(2-ethyl-5-oxohexyl) phthalate; MECCP: Mono-(2-ethyl-5-carboxypentyl) phthalate; MnBP: Mono-n-butyl phthalate; MBzP: Mono-benzyl phthalate; MCOP: Mono-carboxyoctyl phthalate; MCNP: Mono-carboxyisononyl phthatlate; MCPP: Mono-(3-carboxypropyl) phthalate.

**Table 5:** Association (OR (95% CI)) between urinary concentration of phthalate metabolites and atopic dermatitis according to the presence of elevation of total IgE (multivariate model).

Quartile	Urinary Phthalate Metabolites							
	MEHHP	MEOHP	MECCP	MnBP	MBzP	MCOP	MCNP	MCPP
Atopic dermatitis								
1(ref)	1	1	1	1	1	1	1	1
2	0.72	0.97	1.07	0.92	1.33	1.05	0.94	1.02
	(0.40–1.27)	(0.63–1.48)	(0.70–1.65)	(0.52–1.65)	(0.74–2.38)	(0.64–1.71)	(0.58–1.53)	(0.59–1.75)
3	0.98	0.75	0.92	0.76	1.15	1.49	1.07	1.03
	(0.56–1.73)	(0.43–1.33)	(0.54–1.56)	(0.45–1.31)	(0.71–1.87)	(0.88–2.55)	(0.65–1.74)	(0.65–1.63)
4	1.38	1.37	1.81	1.17	1.81	1.32	1.05	1.15
	(0.86–2.22)	(0.85–2.22)	(1.16–2.80)	(0.66–2.08)	(1.01–3.25)	(0.77–2.26)	(0.66–1.66)	(0.71–1.86)
Atopic Dermatitis with Elevation of Total IgE								
1(ref)	1	1	1	1	1	1	1	1
2	0.71	1.17	0.77	0.75	1.30	1.18	1.21	0.93
	(0.37–1.34)	(0.69–1.97)	(0.45–1.31)	(0.40–1.39)	(0.67–2.52)	(0.69–2.00)	(0.63–2.31)	(0.52–1.65)
3	0.64	0.69	0.64	0.82	0.89	1.39	1.39	1.38
	(0.37–1.10)	(0.35–1.37)	(0.30–1.36)	(0.44–1.53)	(0.51–1.56)	(0.78–2.46)	(0.77–2.51)	0.84–2.26)
4	1.36	1.47	1.24	1.19	1.91	1.16	1.11	1.27
	(0.83–2.23)	(0.86–2.52)	(0.77–1.99)	(0.65–2.17)	(1.04–3.48)	(0.64–2.08)	(0.63–1.97)	(0.79–2.05)

Adjusted for gender, school grade, household income, secondhand smoke, and BMI. MEHHP: Mono-(2-ethyl-5-hydroxyhexyl) phthalate; MEOHP: Mono-(2-ethyl-5-oxohexyl) phthalate; MECCP: Mono-(2-ethyl-5-carboxypentyl) phthalate; MnBP: Mono-n-butyl phthalate; MBzP: Mono-benzyl phthalate; MCOP: Mono-carboxyoctyl phthalate; MCNP: Mono-carboxyisononyl phthatlate; MCPP: Mono-(3-carboxypropyl) phthalate.

## Data Availability

The data that support the findings of this study are available after the consideration of the raw data request form by the National Institute of Environmental Research, Environmental Health Research Department, http://meta.narastat.kr/metasvc/svc/SvcMetaDcDtaPopup.do?confmNo=106027&inputYear=2017 (accessed on 10 December 2020).
